# Nanoscale Spatially Resolved Mapping of Uranium Enrichment

**DOI:** 10.1038/s41598-019-48479-5

**Published:** 2019-08-23

**Authors:** Elizabeth Kautz, Douglas Burkes, Vineet Joshi, Curt Lavender, Arun Devaraj

**Affiliations:** 1 National Security Directorate, Pacific Northwest National Laboratory, 902 Battelle Boulevard, P.O. Box 999, Richland, WA 99354 United States; 2 Energy and Environment Directorate, Pacific Northwest National Laboratory, 902 Battelle Boulevard, P.O. Box 999, Richland, WA 99354 United States; 3 Physical and Computational Sciences Directorate, Pacific Northwest National Laboratory, 902 Battelle Boulevard, P.O. Box 999, Richland, WA 99354 United States

**Keywords:** Metals and alloys, Nuclear fuel, Characterization and analytical techniques

## Abstract

Spatially resolved analysis of uranium (U) isotopes in small volumes of actinide-bearing materials is critical for a variety of technical disciplines, including earth and planetary sciences, environmental monitoring, bioremediation, and the nuclear fuel cycle. However, achieving subnanometer-scale spatial resolution for such isotopic analysis is currently a challenge. By using atom probe tomography—a three-dimensional nanoscale characterisation technique—we demonstrate unprecedented nanoscale mapping of U isotopic enrichment with high sensitivity across various microstructural interfaces within small volumes (~100 nm^3^) of depleted and low-enriched U alloyed with 10 wt% molybdenum that has different nominal enrichments of 0.20 and 19.75% ^235^U, respectively. We map enrichment in various morphologies of a U carbide phase, the adjacent γ-UMo matrix, and across interfaces (e.g., carbide/matrix, grain boundary). Results indicate the U carbides were formed during casting, rather than retained from either highly enriched or depleted U feedstock materials. The approach presented here can be applied to study nanoscale variations of isotopic abundances in the broad class of actinide-bearing materials, providing unique insights into their origins and thermomechanical processing routes.

## Introduction

Uranium (U) is the heaviest element naturally occurring in the Earth’s crust in significant amounts, and is used in its natural and anthropogenic forms. U isotopes are relevant to a diverse set of scientific disciplines, most notably earth and planetary sciences^1–8^, toxicology, environmental monitoring and bioremediation^9–11^, the nuclear fuel cycle, forensics, and safeguards^12–22^. Specifically, in nuclear fuel cycle applications, the amount of the fissionable U isotope (^235^U) relative to the total U (i.e., the enrichment) is a critical parameter in fuel performance, and thus affects economic viability of nuclear power. Enrichment of U beyond its natural abundance of 0.7% is necessary to allow a self-sustaining fission chain reaction to proceed in a nuclear fuel^23^. The distribution of ^235^U in a nuclear fuel before irradiation can directly influence the nucleation and distribution of fission products and fission gas bubbles, which affects fuel swelling kinetics^24^. Fuel swelling is directly related to geometric stability (e.g., bowing, deflection) and robustness of fuel plates under irradiation, contributing to phenomena such as coolant channel closure, or release of fission gas products to the coolant^25^. These phenomena make it critical to analyse the distribution of ^235^U in a nuclear fuel at a high spatial resolution to account for ^235^U enrichment variation across all possible nanoscale heterogeneities in the microstructure. Further, activity ratio (e.g., ^234^U to ^238^U or ^234^U to ^235^U, where ^234^U is a decay product of ^238^U) is another critical measure that can provide key insight into sample age and history. For example, activity ratios in naturally occurring U can help distinguish anthropogenic versus naturally occurring forms of U^26,27^. The small volume (nanometres to micrometres in diameter) of precipitates or interfacial regions that must be analysed limits the ability of many bulk analysis techniques currently used for quantifying ^235^U enrichment, introducing a crucial technological gap that prolongs the related knowledge gap. Our work aims to address this gap, and to also demonstrate a methodology by which ^235^U isotopic abundance can be measured quantitatively with subnanometer-scale spatial resolution to gain uniquely powerful insight into material origin, or processing history.

 A primary goal of the United States High Performance Research Reactor Conversion Program is to replace all the highly enriched uranium (HEU) fuel currently used in research reactors to a low-enriched uranium (LEU) fuel. Development of such a LEU fuel system has significant implications to international nuclear nonproliferation, safeguards, and health and environmental safety due to the risks associated continued handling of HEU fuels^18,25,28–32^. Metallic LEU alloyed with 10wt% molybdenum (LEU-10Mo) with less than 20% ^235^U enrichment is a leading candidate for this effort. As a part of fuel qualification and testing, multiple batches of LEU alloy fuel plates (e.g., U-Mo, U-Si, U-Al) have been fabricated and irradiated in research reactors. The microstructures of these irradiated fuels have also been examined after neutron irradiation in order to improve understanding of material behaviour in a reactor operating environment^25,33,34^. Microstructural features observed in irradiated fuels via optical and electron microscopy techniques range from large fission gas bubbles to inclusions and elemental segregation. To develop a comprehensive, atomic-level understanding of fuel performance in a reactor operating environment, it is critical to understand distribution of the fissionable ^235^U isotope in the starting fuel microstructure, at a nanoscale spatial resolution, to determine the isotopic distribution in different phases and across interfaces.

Measurement of U enrichment in small volumes of actinide-bearing materials at nanoscale spatial resolution is also crucial to a variety of other fields beyond nuclear energy. For example, environmental monitoring and bioremediation efforts in the wake of nuclear accidents (such as Fukushima Daiichi, Three Mile Island, or Chernobyl) and detonation of nuclear weapons (for testing or in wartime) depend on detection and mapping of actinide species, particularly U. As part of the environmental monitoring process, measurement of U concentration in geological materials (e.g., soil) or particulate matter (produced from a detonation event) is performed. The measurement of U isotopic abundances can improve understanding of atomic-scale transport of radionuclides in natural materials, the origin of the particles (including age and processing history), how radioactive particles spread, and how harmful they are to the surrounding environment and communities^9,10,22^. Another scientific area where U isotopic enrichment measurement is critical is in regulating the international transportation of actinide-bearing materials. Isotopic measurements in nuclear materials and comparison to existing models is necessary for understanding sample history and ensuring safe handling^14,21,22^. High-spatial-resolution analysis of U enrichment of small volumes is essential to this wide variety of scientific areas.

Current capabilities for measurement of U isotopic abundance typically involve mass spectrometry or spectroscopy methods, including the following: inductively coupled plasma mass spectrometry (ICP-MS)^35^, time-of-flight and nanoscale secondary ion mass spectrometry (SIMS)^36,37^, thermal ionisation mass spectrometry (TIMS)^35^, and gamma spectroscopy^38^. These techniques each have their own merits, and all vary in terms of accuracy, resolution (e.g., spatial, depth, mass), cost, sample preparation requirements, analysis time, and complexity of operation^39^. Each of these considerations is important when selecting the appropriate technique for U isotopic measurements for various purposes. ICP-MS is routinely used due to simple sample preparation, short measurement times, and high sensitivity for even very low U isotopic ratios (e.g., ^236^U/^238^U ratios on the order of ~10^−7^–10^−8^, or 0.0001–0.00001% ^236^U)^40^. However, since the analysis is done in the gas phase for ICP-MS, spatial mapping within the sample is not possible. NanoSIMS does offer the advantage of spatial mapping of isotopic abundances with lateral spatial resolution of 50 nm, but does not have equal sensitivity for different phases^36^, Further, gamma spectroscopy is a very common, nondestructive method that could provide mapping of isotopic abundances over a large area, but with relatively low spatial resolution (~millimetres). The major gaps existing in techniques currently used for U isotopic abundance measurements are the combination of high spatial and mass resolution, and the ability to probe isotopic abundances in nanoscale volumes and across interfaces. However, these techniques cannot provide the subnanometer-scale spatial resolution required to probe fine-scale microstructural features. Atom probe tomography (APT) is a 3D nanoscale characterisation method uniquely suited to analyse both composition and isotopic abundances in various material systems, with high spatial resolution^41–43^. The high resolution available via APT is increasingly important for many application areas, for example in nuclear forensics, geochronology, and environmental remediation, where analysis of nanoparticles could provide key insights into origin or processing history. Additionally, for new nuclear fuel material design, nanoscale inclusions (i.e., secondary phases) must be analysed within a bulk fuel sample to better design fuel processing and predict material performance in a neutron irradiation environment. The only technique that can probe such nanoscale volumes and still achieve high spatial and mass resolution is APT.

Our work demonstrates an approach for using APT to obtain nanoscale, spatially resolved, quantitative ^234^U, ^235^U, and ^238^U isotopic mapping, which has not been demonstrated before in anthropogenic U-bearing materials. We selected metallic U-10Mo nuclear fuels with two different enrichment values for demonstrating this capability^44–46^.

## Results

### 3D elemental distribution across precipitate-matrix interfaces

**Figure 1:**
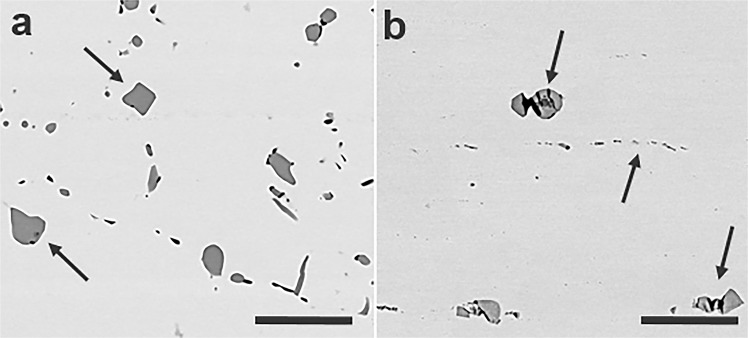
Microstructures of depleted and low-enriched U-10Mo alloys: Backscattered scanning electron microscope images of microstructural features in the nuclear fuel analysed via APT in (**a**) DU-10Mo and (**b**) LEU-10Mo. The arrows point towards carbides. Two distinct carbide morphologies are visible in these micrographs: coarse and fine. The scale bar in each micrograph is 20 µm long.

In both depleted U-10Mo (DU-10Mo) and LEU-10Mo alloys, the main microstructural features are uranium carbide (UC) inclusions and the surrounding γ-UMo matrix^44^. Representative images of both DU-10Mo and LEU-10Mo microstructures are shown in Fig. 1. (Example micrographs of other UC morphologies are provided in Fig. S1 of the Supplementary Information). The UC phase is distributed throughout the γ-UMo matrix in both alloys, and was found to have two distinct morphologies in LEU-10Mo: (1) fine, i.e., approximately 500 nm wide, and (2) coarse, approximately 3–5 µm wide. Both UC morphologies, in addition to the γ-UMo matrix phase, were analysed via APT in order to determine U enrichment and elemental distribution in both phases and across γ-UMo matrix/UC interfaces.

**Figure 2:**
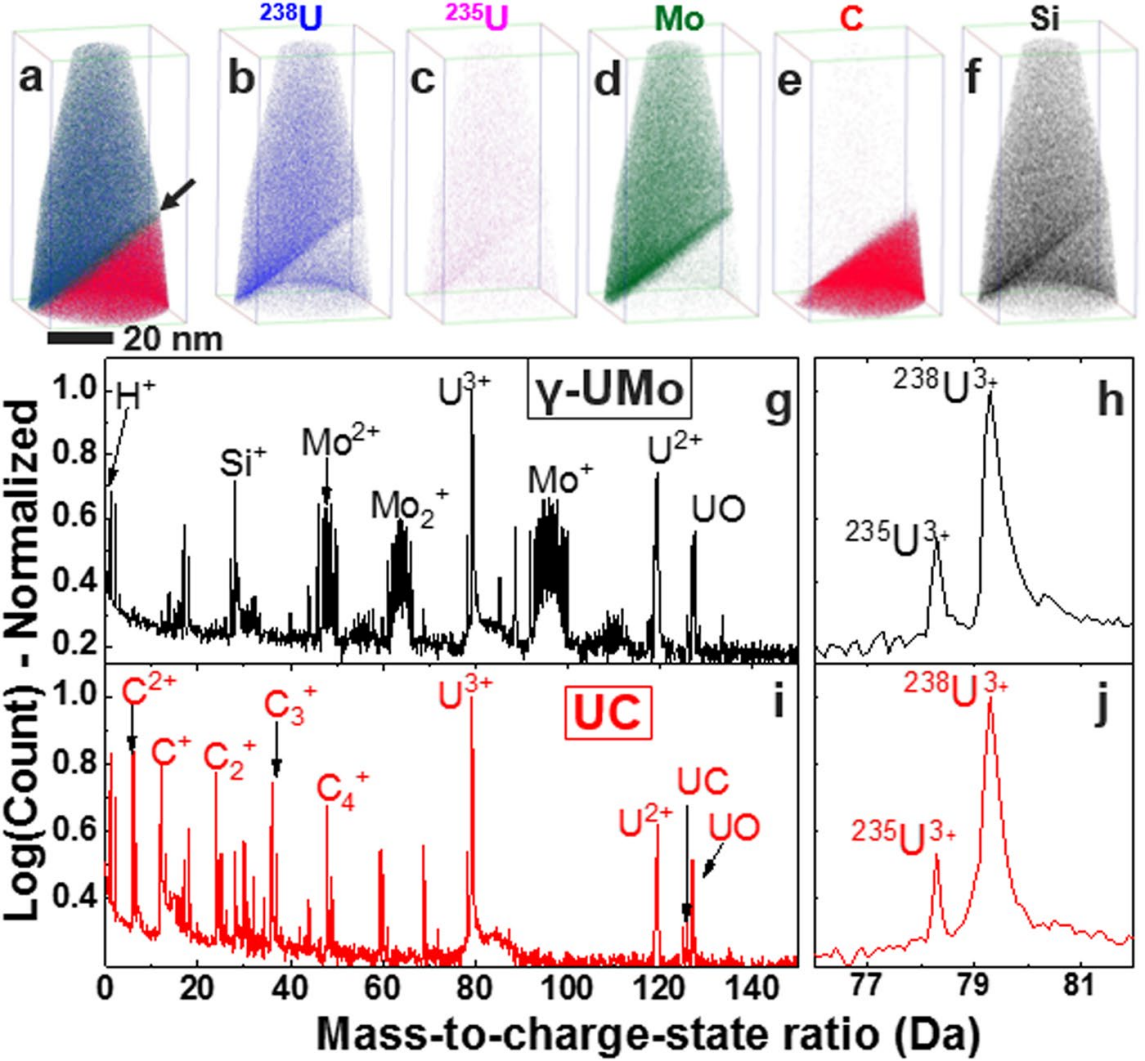
APT results for a depleted U-10Mo alloy: (**a**–**f**) 3D element distribution maps of all detected ions, U isotopes (^235^U, ^238^U), Mo, C, and Si across a DU-10Mo/UC interface, which is indicated by the black arrow in (**a**). Full mass spectra (0–150 Da) for (**g**) γ-UMo matrix and (**i**) UC phase. Specific region of the mass spectra between 76 and 82 Da showing the U^3+^ peaks used for isotopic abundance calculations for the γ-UMo matrix (**h**) and UC phases (**j**). Mass spectra shown in (**g**–**j**) are for a 0.1 nm fixed bin width.

**Figure 3:**
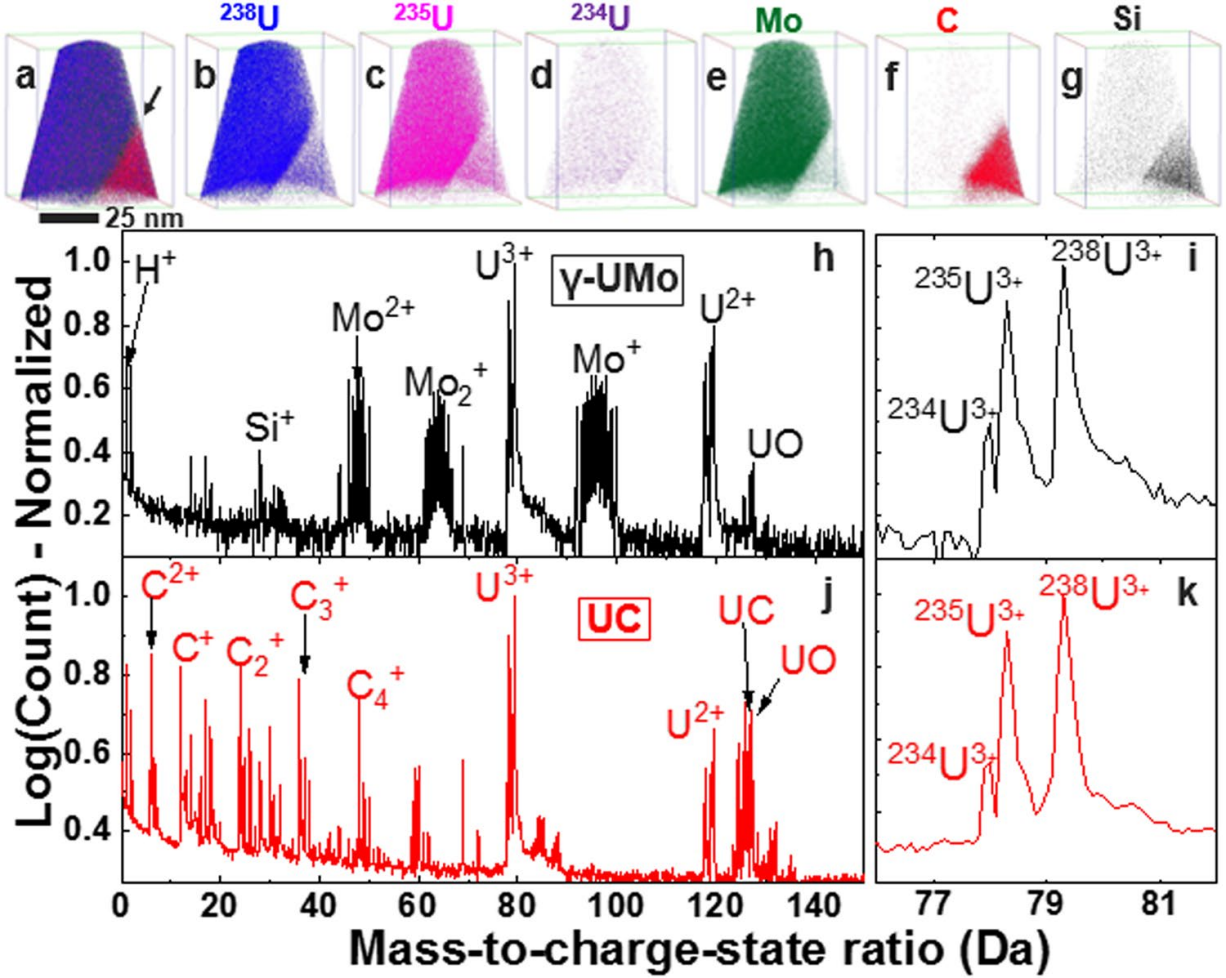
APT results for a low-enriched U-10Mo alloy: (**a**–**g**) 3D element distribution maps of all detected ions, U isotopes (^234^U, ^235^U, ^238^U), Mo, C, and Si across a LEU-10Mo/UC interface, which is indicated by the black arrow in (**a**). Full mass spectra (0–150 Da) for (**h**) γ-UMo matrix and (**j**) UC phase. Specific region of the mass spectra between 76 and 82 Da showing the U^3+^ peaks used for isotopic abundance calculations for the γ-UMo matrix (**i**) and UC phases (**k**). Mass spectra shown in (**h**–**k**) are for a 0.1 nm fixed bin width.

3D distributions of major alloying elements (U, Mo) and impurity elements (Si, C) from DU-10Mo and LEU-10Mo alloys are given in Figs 2 and 3, respectively, with corresponding mass spectra for γ-UMo and UC phases. Mass spectra were normalised to the peak with the maximum number of counts, which corresponds to the ^238^U^3+^ peak in all data sets collected as part of this work. Normalised mass spectra were generated using manual ranging criteria. For each phase, U^3+^ peaks are shown in the portion of the mass spectra between mass-to-charge-state ratios of 76 and 82 Da (daltons). For DU-10Mo, ^235^U^3+^ and ^238^U^3+^ peaks were detected, and in the LEU-10Mo alloy, the ^234^U^3+^ peak was also visible in the spectra; ^234^U is known to be a decay product of ^238^U. In depleted U, ^234^U should account for only approximately 0.001% of all U isotopes (10 ppm), which is approaching the detection limit of APT^41^. These results highlight the capability of APT to resolve each U isotopic peak individually; the peaks also can be spatially resolved in the 3D reconstruction.

U and Mo were observed as the major species in both DU-10Mo and LEU-10Mo samples, where ^235^U and ^238^U were found to field evaporate as doubly (U^2+^) and triply (U^3+^) charged ions. Complex ions composed of U and H (UH, or uranium hydride) were also present in mass spectra of both alloys. UH peaks were detected at approximately 118.18 and 119.66 Da (corresponding to the U^2+^ charge states for ^235^U and ^238^U, respectively) in LEU-10Mo. In DU-10Mo, however, only the peak at 119.66 Da was observed. The mass spectra for U^2+^ for both alloys analysed are provided in the Supplementary Information, Fig. S2, and show all detected UH peaks.

**Figure 4:**
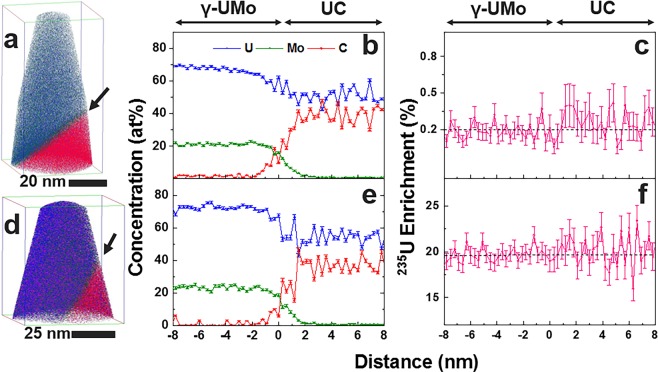
Compositional and isotopic profiles of DUMo and LEUMo. (**a**) 3D element distribution map that contains a γ-UMo/UC interface in a DU-10Mo alloy, (**b**) corresponding compositional profile across the matrix/UC interface, (**c**) map of ^235^U% relative to all U isotopes (enrichment) across the γ-UMo/UC interface in the DU-10Mo. Panels (d–f) present the same type of data provided in (**a**–**c**), but for a LEU-10Mo alloy. Arrows in (a) and (d) point towards the γ-UMo/UC interface. Composition profiles were calculated for a 0.3 nm fixed bin width. Error plotted in (**b**,**c**,**e**,**f**) is calculated based on point-counting error.

The changes in concentration of U and Mo and other impurity elements C and Si were quantified using a one-dimensional (1D) concentration profile across the γ-UMo matrix/UC interface (Fig. 4). Peak deconvolution was performed to determine total U, Mo, C, and Si contributions from both elemental and complex peaks present in the mass-to-charge spectrum, a process further detailed in the Materials and Methods section. U and Mo concentrations were consistent with the composition of U alloyed with 10 wt% Mo (approximately 22 at% Mo) for the γ-UMo matrix phase. In the γ-UMo matrix, C and Si impurity elements were also detected at concentrations of less than 3 at%. UC compositions measured by APT were 51–54 at% U and 35–39 at% C. In the UC phase, concentrations of impurity elements (Si, H, O, Ni, Al) account for the balance. Analysis of impurity elements including Si, H, O, Ni, Al was also performed, and concentrations of these elements were plotted across the matrix/carbide interface in DU-10Mo and LEU-10Mo alloys; these are reported in the Supplementary Information, Fig. S3. The concentration of Si was less than 3 at%, and those of O, Ni, and Al were all less than 2 at% in samples analysed, whereas H concentration was more significant, and was found in concentrations of 4–20 at%. The H concentration can be attributed to the formation of UH when U is in the presence of H^17^. Sources of H leading to the formation of UH could be water used in the bulk metallographic polishing procedure, focused ion beam (FIB) preparation of APT needle specimens, and/or residual H from the APT analysis chamber.

### Quantification of U enrichment in individual phases

Uranium isotope abundances in γ-UMo phases and in fine and coarse morphologies of UC were quantified from APT data, and the results are summarised in Table 1. U isotopic enrichment was estimated based on the following equation:

$${U}_{enrichment} \% =\frac{{U}_{i} \% }{{U}_{total} \% }\times 100$$

Where *U*_*i*_% corresponds to the atomic percentage of a particular U isotope and *U*_*total*_% corresponds to the total U atomic percentage estimated from APT results, accounting for all U isotopes present in the spectrum. Sample volumes from which U isotopic abundances were calculated ranged from 8,000 to 250,000 nm^3^, corresponding to volumes with dimensions of ~20 nm × 20 nm × 20 nm to 50 nm × 50 nm × 100 nm. U ion counts from the U^3+^ elemental peaks accounted for the majority of U ions collected; thus, isotopic ratios were calculated using this peak count. The percentages of U elemental and U complex species (e.g., UC, UH, UCH) are reported in the Supplementary Information, Table S1, and a comparison of calculated U enrichment from different charge-state peaks are provided in Table S2. From APT results we did not observe any U isotopic bias regarding preferential evaporation as molecular ions, which was more prevalent in the UC phase. Thus, calculating isotopic abundances based on the major elemental peak (U^3+^) provided the most consistent and accurate approach for analysis.

**Table 1:** U isotopic abundances measured by APT: Tabulated results are from different phases from both depleted and low-enriched U-10Mo alloys.

Alloy	Phase	Percent ^235^U	Percent ^238^U	Percent ^234^U
DU-10Mo	γ-UMo matrix	0.19 ± 0.001	99.81 ± 0.060	—
		0.19 ± 0.005	99.81 ± 0.208	—
	UC - coarse	0.21 ± 0.017	99.79 ± 0.676	—
		0.20 ± 0.016	99.79 ± 0.673	—
		0.21 ± 0.005	99.79 ± 0.210	—
LEU-10Mo	γ-UMo matrix	19.47 ± 0.185	80.34 ± 0.527	0.189 ± 0.015
		19.26 ± 0.078	80.51 ± 0.222	0.226 ± 0.007
	UC - coarse	20.44 ± 0.051	79.34 ± 0.138	0.218 ± 0.004
		20.66 ± 0.029	79.11 ± 0.078	0.233 ± 0.003
		20.17 ± 0.034	79.61 ± 0.093	0.219 ± 0.003
	UC - fine	20.80 ± 0.544	78.97 ± 1.623	0.229 ± 0.053
		20.00 ± 0.042	79.79 ± 1.075	0.211 ± 0.003
		20.04 ± 0.051	79.74 ± 0.141	0.226 ± 0.005

All data in this table are reported as percentage of U isotope relative to all U with percent error, where percentages were calculated based on ion count of each isotope. ^234^U% is not reported for DU-10Mo and is assumed to be less than 0.001%. Error was calculated by propagating point-counting error, which is subsequently detailed in the Materials and Methods section. For each phase, measurements from multiple APT needles are reported.

In Table 1, percentages of ^235^U and ^238^U are reported for DU-10Mo, and percentages of ^234^U, ^235^U, and ^238^U are reported for LEU-10Mo. The percentage of ^234^U in DU-10Mo is expected to be approximately 0.001%, as previously discussed. No distinct ^234^U peak in the mass spectra was observed for DU-10Mo samples (as demonstrated in Fig. 2), and thus the amount of ^234^U present is assumed to be below the detection limit of APT. In all LEU-10Mo samples analysed, there was a clear ^234^U peak (as previously reported in Fig. 3); measured abundances were much greater than the 0.001% detection limit, and were therefore assumed to be accurate estimates of ^234^U % even at amounts less than 0.3%.

### Analysis of U enrichment across a γ-UMo grain boundary

**Figure 5:**
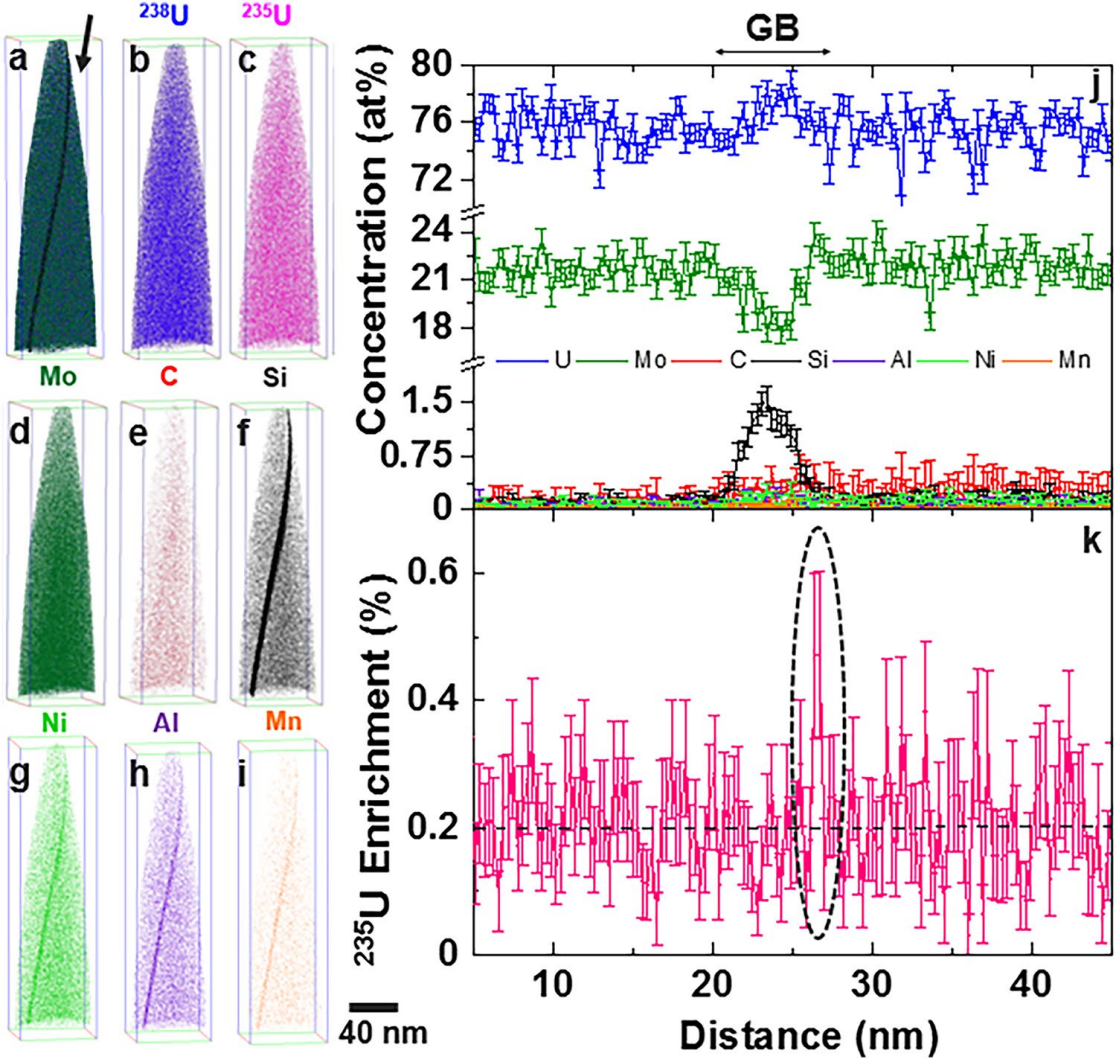
Compositional and isotopic profiles of a DU-10Mo grain boundary. (**a**–**i**) 3D element distribution maps for major alloying and impurity elements; (**j**) corresponding quantitative analysis of composition across the grain boundary (concentration in at% versus distance) via a 1D concentration profile; (**k**) ^235^U enrichment across the grain boundary. Error plotted in (**j**,**k**) is calculated based on point-counting error. The black arrow in (a) points towards the grain boundary analyzed.

Grain boundaries in metallic nuclear fuels play an important role in material behaviour in a reactor operating environment. Grain boundaries directly influence recovery from radiation damage and accumulation of defects, such as fission gas bubble nucleation and growth in irradiated fuel plates^25^. Hence, as a proof-of-principle demonstration, APT was additionally used to analyse variation of isotopic enrichment across the γ-UMo grain boundary in the DU-10Mo alloy. The APT reconstruction across a γ-UMo grain boundary in DU-10Mo is given in Fig. 5 along with the compositional profile showing concentration of major alloying elements (U, Mo), impurity elements (Si, C, Ni, Al, Mn), and ^235^U enrichment. There is clear solute segregation along the grain boundary, consistent with our prior work^44^. Interestingly, there is a narrow band of increase in ^235^U enrichment specifically at the grain boundary (highlighted by the dashed black line in Fig. 3(k)) where locally the enrichment increased to as high as 0.5%, while the overall enrichment in either grain remained consistent with the nominal value of ~0.2% ^235^U. This result highlights the unique capability of APT to not only accurately probe the compositional segregation across grain boundaries in metallic nuclear fuels, but also obtain accurate information on local variation in ^235^U enrichment across grain boundaries.

## Discussion

Characterisation of UC and γ-UMo matrix phases in DU and LEU alloyed with 10 wt% Mo was performed to demonstrate the capability of APT for quantitative measurement of U isotopes in actinide-bearing materials with varying nominal enrichments. The purpose of this effort was to gain insight into the homogeneity of ^235^U across different microstructural features, which has implications for fuel swelling and thus stability of nuclear fuel in a reactor operating environment. The high 3D spatial resolution achievable is a unique features of APT that other techniques more routinely used for such analyses (e.g. ICP-MS, gamma spectroscopy, SIMS) do not provide.

Calculated ^235^U isotopic abundances for both DU-10Mo and LEU-10Mo (summarised in Table 1) indicate that U isotopic abundances are comparable between matrix and carbide phases, with minor fluctuations (of less than 1%) of U enrichment reported. This result suggests that fuel fabrication processes generate a microstructure in which ^235^U is relatively homogeneously distributed between carbide and matrix phases. The low enrichment of carbides measured by APT indicate that the analysed carbides likely formed during the melting and casting processes and were not retained from the HEU or DU feedstock materials that were melted together and cast to form the LEU-10Mo alloy. The work presented here demonstrates that it is possible to probe U isotope abundances in fine microstructural features and across interfaces in nuclear fuels to establish microstructure-processing relationships. In order to qualify APT for analysing differences of <1% in U enrichment, further detailed investigation of the influence of user-selected APT experimental parameters (e.g., laser-assisted versus voltage pulsing modes, pulse frequency, laser pulse energy, specimen base temperature, detection rate) and difference in field evaporation behaviour between phases is warranted. Our results allowed us to gain insight into the sample processing history; the concept and approach can now be applied to forensics and environmental remediation studies and surpass current understanding based on other mass spectrometry and spectroscopy techniques. Additionally, the result that ^235^U is nominally homogeneously distributed at the nanoscale between the γ-UMo matrix and UC phases can inform fuel performance modelling.

Impurity elements (C, Si) were measured via APT, and some local increase in Si concentration was observed along the UC/γ-UMo interface in DU-10Mo. A higher concentration of Si was observed in the UC phase of the LEU-10Mo alloy in comparison to UC phase in DU-10Mo alloy. From the U isotope abundances given in Table 1, it is clear that APT can measure not only the major isotopes ^235^U and ^238^U, but also ^234^U. The APT reconstructions shown in Figs 2 and 3 from DU-10Mo and LEU-10Mo, respectively, reveal the ability to spatially resolve the isotope enrichment to a spatial resolution of 0.2 nm in *x*, *y* and *z* directions. APT is also uniquely suited for analysis of enrichment across grain boundaries in nuclear fuels, which is a challenging task for most of the bulk isotopic analysis methods routinely used (e.g., ICP-MS, SIMS, gamma spectroscopy). Given this demonstration, our approach can now be used to analyse small particulates from nuclear accidents or at different stages of the nuclear fuel cycle to help analyse U enrichment and identify material origin, age, or processing history. This study opens opportunities to use APT as a method of choice for a variety of other fields ranging from earth and planetary sciences to bioremediation, toxicology, and environmental monitoring. Further, application of APT to isotopic abundance measurements can be used in combination with other technique(s) to compare results and understand over what length scales isotopes are homogeneously distributed.

In summary, by using site-specific sample preparation and APT analysis, we demonstrate the unique capability to perform spatially resolved nanoscale mapping of isotopic enrichment and impurity elements in U-Mo fuels with two different ^235^U enrichments. Based on the results reported herein, we believe APT has a strong prospect for use in a wide range of characterisation efforts of actinide-bearing materials (e.g., U) in which isotopic analysis in small volumes (and of specific microstructural features) is crucial. Our work has implications for multiple disciplines that could benefit from precise measurement of U isotope enrichment and fractionation to better understand sample origin or processing history. The methods and experimental protocols using APT presented here translate well to analysis of U enrichment in many other systems (geologic materials, metallic fuels, and glass fabricated as a nuclear waste form, for example). Thus, APT is promising as an effective and versatile tool for measurement of isotopic abundances in actinide-bearing materials, at length scales previously unexplored by other more widely used mass spectrometry and spectroscopy methods.

## Materials and Methods

### Material fabrication

DU-10Mo and LEU-10Mo fuels analysed in this work were fabricated at the Y12 National Security Complex at Oak Ridge National Laboratory. DU-10Mo samples were fabricated by melting DU with Mo and casting in a graphite mould. The cast DU-10Mo was then homogenised at 900 °C for 48 hours in an inert argon (Ar) gas atmosphere. After the homogenisation anneal, samples were cooled by turning off the furnace and flowing Ar gas into the chamber to help cool the samples. A cooling rate of 25 °C per minute was achieved from 900 °C to 650 °C, then 3 °C per minute from 500 °C to 350 °C. LEU-10Mo samples were homogenised at 900 °C for 144 hours in the same atmosphere and furnace as previously described. The LEU-10Mo alloy was subjected to subsequent thermomechanical processing treatments (hot and cold rolling) to form the final fuel foil. Additional sample fabrication details are reported elsewhere^44,45^.

### FIB-based sample preparation and APT analysis

APT needles were prepared from standard metallographically polished bulk samples^47^. Site-specific FIB lift-outs and annular milling were performed to shape specimens into needles for APT analysis^41^.

A CAMECA LEAP 4000X HR APT system equipped with a 355 nm wavelength (UV) laser was used for all APT data collection with the following user-selected parameters: 100 pJ laser energy, 100 kHz pulse frequency, 45 K specimen temperature, and 0.005 atoms/pulse detection rate. Data sets analysed and reported here ranged in size between 1.4 and 34.5 million ions, and either contain a single phase (UC or γ-UMo matrix) or captured a UC/γ-UMo interface. Additionally, a data set from the DU-10Mo alloy contained a grain boundary. All data sets were reconstructed and analysed using the Interactive Visualization and Analysis Software (IVAS), version 3.8.2. For data sets containing a single phase, bulk composition analysis was completed, and for those data sets that contained an interface, composition in each phase was determined using spherical regions of interest (ROIs) with diameters of 20–30 nm.

To examine composition across an interface, a cylindrical ROI with a diameter of 20 nm was positioned perpendicular to the planar interface, and a 1D composition profile along the *z*-axis with a 0.3 nm bin width was constructed. For all composition analysis, ionic and background-corrected data were used.

In order to quantify Mo and C concentrations accurately from raw data, peak deconvolution was performed^48^. Peak deconvolution was required to distinguish between ^96^Mo^2+^ and C_4_ species, which both have a mass-to-charge-state ratio of approximately 48 Da, and therefore cannot be ranged separately. The number of ^96^Mo^2+^ ions was estimated from the number of ^98^Mo^2+^ ions, assuming both ^96^Mo^2+^ and ^98^Mo^2+^are present in U-Mo at naturally abundant levels, similar to the process reported in work by Taylor *et al*.^48^. Equation (1) was used to determine the ^96^Mo^2+^ ion count:

1 $${n}_{{}^{96}M{o}^{2+}}={n}_{{}^{98}M{o}^{2+}}\times \frac{0.1668}{0.2413}$$

where ${n}_{{}^{96}M{o}^{2+}}$ is the estimated ion count for the ^96^Mo^2+^, and ${n}_{{}^{98}M{o}^{+2}}$ is the ion count for the ^98^Mo^2+^ peak. The two fractions in equation (1) (0.1668 and 0.2413) are the natural isotopic abundances of ^96^Mo and ^98^Mo, respectively. Since the entire ^96^Mo^2+^ peak was ranged as a single species but not all counts correspond to ^96^Mo^2+^, the calculated value of ${n}_{{}^{96}M{o}^{2+}}$ was subtracted from the total ion counts corresponding to the ranged peak to obtain the ion count for the C_4_ species.

In order to obtain an accurate estimation of C concentration in the volume analysed, we used the ^13^C method^49^. This approach involves multiplying the ^13^C background-corrected ion count by 92.5 to determine a more accurate value for the ^12^C ion count. The 92.5 multiplier relies on the assumption that C exists in naturally abundant levels.

Peak deconvolution was performed via methods described above to analyse the total U, Mo, C, and Si concentrations across the interface. Error reported in tables and composition profiles represents propagation of point-counting error, defined as

2 $${E}_{i}=\sqrt{\frac{{C}_{i}(1-{C}_{i})}{N}}$$

where *C*_*i*_ is the number of solute ions of element i, *N* is the total number of ions in a bin, and *x*_*i*_ is the count for a specific element. *C*_*i*_ is defined in mathematical form as

3 $${C}_{i}=\frac{{x}_{i}}{N}$$

Error reported as a percentage is simply the value calculated from Eq. (2) multiplied by 100. Error associated with U enrichment was calculated with Eqs (4, 5), where *E*_*Total*,*U*_ (Eq. (4)) is the total error associated with all U isotope counts, and ${E}_{{U}_{enrichment}}$ (Eq. (5)) is the counting error associated with a U isotope enrichment value. In Eq. 5, *U*_*i*_ is the total ion count for a particular isotope, *E*_*Ui*_ is the point counting error for that isotope, *E*_*Total,U*_ is defined in Eq. 4, and *U*_*total,conc*_ is the total count for all U isotopes. Eq. 5 represents the propogation of point counting error for enrichment error calculation.

4 $${E}_{{T}{o}{t}{a}{l},U}=\sqrt{{({E}_{{}^{234}U})}^{2}+{({E}_{{}^{235}U})}^{2}+{({E}_{{}^{238}U})}^{2}}$$

5 $${E}_{{U}_{enrichment}}={U}_{i}\times \sqrt{{(\frac{{E}_{{U}_{i}}}{{U}_{i}})}^{2}+{(\frac{{E}_{Total,U}}{{U}_{total,conc}})}^{2}}$$

## Supplementary information

Supplementary information
