# Supplementary material for: Nanoscale Spatially Resolved Mapping of Uranium Enrichment
Source: Sci Rep. 2019 Aug 23;9:12302. doi: 10.1038/s41598-019-48479-5 (PMC6707289; doi:10.1038/s41598-019-48479-5)
Supplement: Supplementary file 1 — Supplementary information [file 41598_2019_48479_MOESM1_ESM.docx]

**Supplemental Information**

##### Nanoscale spatially resolved mapping of uranium enrichment

Elizabeth Kautz^a^, Douglas Burkes^a^, Vineet Joshi ^b^, Curt Lavender^b^, Arun Devaraj^c^*

^a^National Security Directorate, Pacific Northwest National Laboratory, 902 Battelle Boulevard, P.O. Box 999, Richland, WA 99352, United States

^b^Energy and Environment Directorate, Pacific Northwest National Laboratory, 902 Battelle Boulevard, P.O. Box 999, Richland, WA 99352, United States

^c^Physical and Computational Sciences Directorate, Pacific Northwest National Laboratory, 902 Battelle Boulevard, P.O. Box 999, Richland, WA 99352, United States

*corresponding author: arun.devaraj@pnnl.gov

**Micrographs**

Additional micrographs illustrating carbide morphologies observed in U-Mo alloys analyzed in this work are provided in Supplementary Figure S1. Both coarse and fine uranium carbide phases are shown in these micrographs. These micrographs are intended to supplement those in the body of the manuscript, where the micrographs here are at different magnifications, and are additional examples or carbide morphologies in the fabricated fuel plates.

| 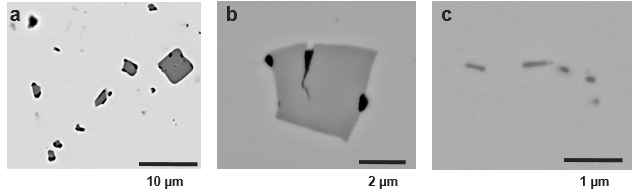 |
| --- |
| **Supplemental Figure S1.** Micrographs of the uranium carbide phase in the γ-UMo matrix of depleted and low-enriched U-Mo alloys characterized via APT. The micrograph in (a) is from the depleted U-10Mo alloy, and (b) and (c) illustrate coarse and fine carbide morphologies respectively, found in a LEU-Mo alloy. The micrographs provided here are higher magnification and additional detail on carbide morphology than those presented in the body of the manuscript. |

**Uranium isotopic abundances calculations**

In order to calculate U enrichment, the question of how to analyze the collected mass spectra was considered in detail.

In this work, the ion counts from the elemental U isotopes in the 3+ charge state were used to calculate percentage of each U isotope. The 3+ charge state ion counts were used for the following reasons: (1) the U^3+^ counts are the majority of U ion counts collected via the APT technique, and (2) no complex peaks needed to be considered (no peak deconvolution required), thus simplifying the data analysis process, and reducing the bias introduced into the analysis procedure.

It is noted here that in order to calculate U isotopic abundances, each peak corresponding to each U isotope detected was ranged separately. Further, each U isotope peak for the 2+ and 3+ charge states were ranged separately. It is noted here that complex ions corresponding to uranium hydride, carbide, and oxide phases were also detected via APT, however these complex ion peaks contributed only a minor amount to overall U ion count, and were thus not considered for use in U enrichment calculations. Further, hydrides are known to be common artefacts in atom probe data, and thus deconvolution of these hydride peaks is non-trivial, and could lead to inaccurate interpretation of isotopic abundances.

Supplementary Table S1 presents a summary of the percentage of different U ions identified in APT mass spectra for matrix and carbide phases for DU-10Mo and LEU-10Mo. Results tabulated in this table indicate that contributions to the U ion count from complex species are minor. Additionally, since there is no evidence for the preferential formation of molecular ions with one isotope versus another of a single species, we assume that by taking into account the isotopic ratios of the major U^3+^ elemental peak, we can accurately calculate enrichment.

Enrichment was calculated using U^2+^ and U^3+^ ions, and is summarized in Supplementary Table S2. Small differences between calculated U enrichments from ion counts from 2+ and 3+ charge states may be due to the more limited number of ions (and thus increased error) used to calculate enrichment from the U^2+^ charge state. These example U enrichment calculations confirm the importance of using the majority ion count in these calculations.

**Supplementary Table S1.** Percent U ion species for U isotopes with 3+ and 2+ charge states for DU-10Mo and LEU-10Mo samples, both matrix and carbide phases. The percentage of complex species is also reported.

| **Charge State** | **Ion Type** | **% U species relative to all U** | | | |
| --- | --- | --- | --- | --- | --- |
|  |  | **γ-UMo matrix** | | **UC** | |
|  |  | **DU-10Mo** | **LEU-10Mo** | **DU-10Mo** | **LEU-10Mo** |
| 3+ | ^238^U | 93 | 72 | 98 | 74 |
|  | ^235^U | <1 | 17 | <1 | 19 |
|  | ^234^U | <1 | <1 | <1 | <1 |
| 2+ | ^238^U | 3 | <1 | <1 | <1 |
|  | ^238^UH | 4 | 8 | <1 | <1 |
|  | ^235^U | <1 | <1 | <1 | <1 |
|  | ^235^UH | <1 | 2 | <1 | <1 |
|  | ^238^UC, ^238^UCH, ^238^UCH_2_, ^235^UC, ^235^UCH, ^235^UCH_2_, ^234^UC, ^234^UCH | <1 | <1 | <1 | <6 |

**Supplementary Table S2.** ^235^U% calculated based on U ion counts from the 2+ charge state, compared to that calculated using the 3+ charge state ions.

|  | | **3+ Charge State** | | **2+ Charge State** | |
| --- | --- | --- | --- | --- | --- |
|  |  | **235-U %** | **Error (%)** | **235-U %** | **Error %** |
| DU-10Mo | γ-UMo matrix | 0.21 | 0.19 | 0.15 | 0.023 |
|  | UC | 0.21 | 0.21 | 0.22 | 0.078 |
| LEU-10Mo | γ-UMo matrix | 19.47 | 0.19 | 18.59 | 1.066 |
|  | UC | 20.44 | 0.05 | 18.49 | 0.766 |

**Uranium hydride peaks**

Example mass spectra from DU-10Mo and LEU-10Mo matrix and carbide phases showing ^235,238^U^2+^, ^235,238^U^2+^H peaks are provided in Supplementary Figure S3.

In all samples analyzed, uranium hydride (UH) peaks were observed next to each U elemental peak in the 2+ charge state. In some cases, a very small U^3+^H peak was observed. The mass spectra shown in Supplemental Figure S3 are intended to be representative of the trends observed in all alloys and phases analyzed.

|  | **γ-UMo matrix** | **UC** |
| --- | --- | --- |
| *DU-10Mo* | 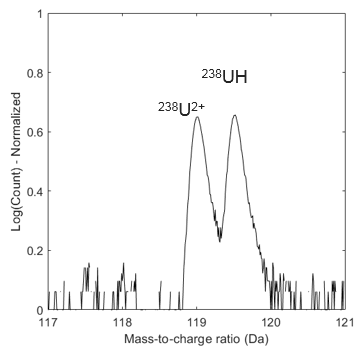 | 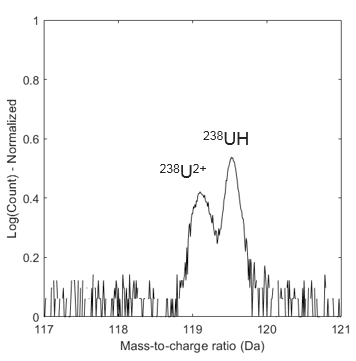 |
| *LEU-10Mo* | 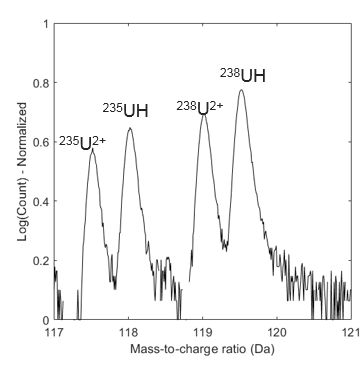 | 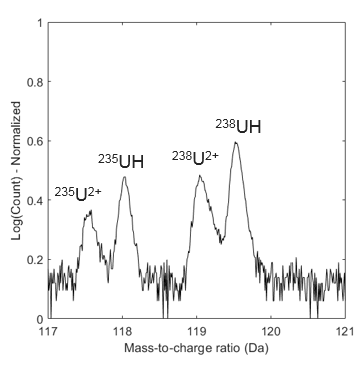 |
| **Supplemental Figure S2.** Mass spectra collected via the local electrode atom probe technique for U-Mo matrix and UC phases in DU-10Mo and LEU-10Mo alloys. All spectra shown here are between 117 and 121 Da. In this range the U^2+^ peak is observed, and a corresponding UH peak. | | |

**Compositional Analysis of Impurity Elements across the γ-U-Mo/UC Interface**

Impurity elements analyzed via atom probe tomography include Si, Ni, Al, O, and H. The composition of these elements (in atom percent, at%) across the γ-U-Mo/UC Interface are plotted in Supplementary Figure 1 for each U-Mo sample analyzed. Manual peak deconvolution was performed in order to quantify element composition.

Hydrogen concentration varied between each sample in comparison to other impurity elements. The trends in H concentration are currently unknown. The source of the H measured in these samples could be attributed to metallographic polishing and residual H in the analysis chamber.

Si concentration is highest for the DU-Mo sample analyzed in comparison to both LEU-Mo samples. The origin of the Si impurity element is hypothesized to be the DU feedstock material. Since different DU samples were used in fabricating LEU-Mo, the starting materials could have been higher purity, but the exact processing to yield this higher purity U-Mo alloys are currently unknown.

Some Ni solute partitioning across the interface was observed in all three samples analyzed, where Ni concentration was observed to be higher in the UC phase versus the matrix. Very low O and Al concentrations were measured via atom probe, although it is noted here that in the DU-Mo bulk sample analyzed, O partitioning was observed across the matrix/UC interface, and the concentration of O in UC was higher than the adjacent matrix.

**UC**

**γ-UMo**

**UC**

**γ-UMo**

| *DU-10Mo* | 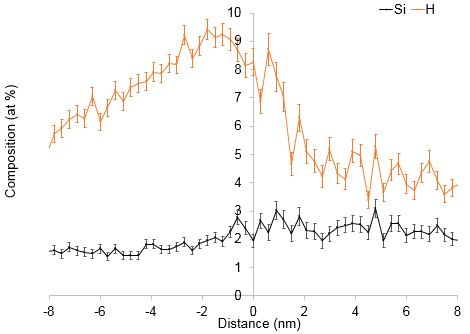 | 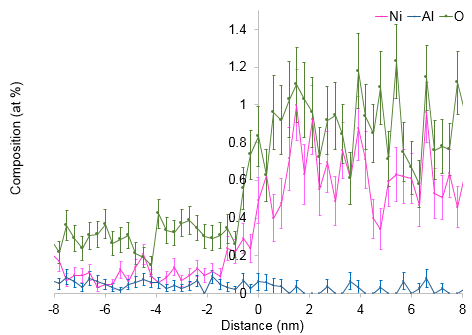 |
| --- | --- | --- |
|  | a | b |
| *LEU-10Mo* | 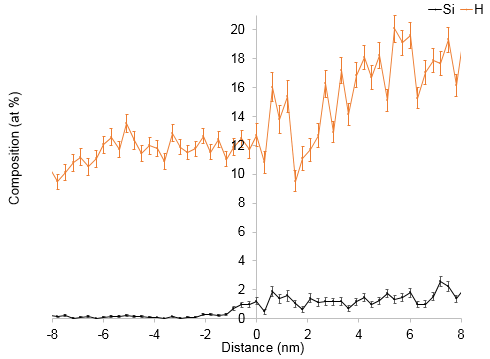 | 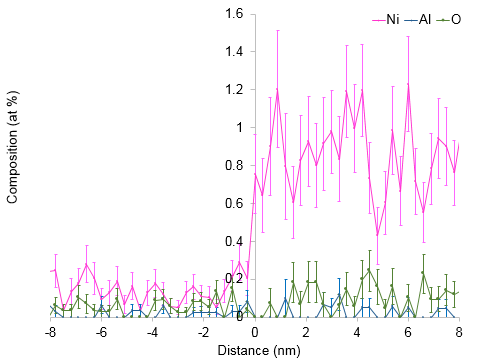 |
|  | c | d |
|  |  |  |
| **Supplementary Figure S3.** Impurity element composition across the gamma-UMo/UC interface for (a,b) DU-10Mo, (c,d) LEU-10Mo. Si and H versus distance are provided in a,c,e, and Ni, Al, and O are provided in b,d,f. All plots show composition (in at%) with error bars corresponding to point counting error for each element. | | |
